# CHD1L maintains genome integrity by facilitating okazaki fragment maturation

**DOI:** 10.1093/nar/gkag606

**Published:** 2026-06-22

**Authors:** Litong Nie, Jialing Fu, Min Huang, Huimin Zhang, Tiantian Ma, Chang Yang, Siting Li, Chao Wang, Junjie Chen

**Affiliations:** Department of Experimental Radiation Oncology, The University of Texas MD Anderson Cancer Center, Houston, TX 77030, United States; Department of Experimental Radiation Oncology, The University of Texas MD Anderson Cancer Center, Houston, TX 77030, United States; Department of Experimental Radiation Oncology, The University of Texas MD Anderson Cancer Center, Houston, TX 77030, United States; Department of Experimental Radiation Oncology, The University of Texas MD Anderson Cancer Center, Houston, TX 77030, United States; Department of Experimental Radiation Oncology, The University of Texas MD Anderson Cancer Center, Houston, TX 77030, United States; Department of Experimental Radiation Oncology, The University of Texas MD Anderson Cancer Center, Houston, TX 77030, United States; Department of Experimental Radiation Oncology, The University of Texas MD Anderson Cancer Center, Houston, TX 77030, United States; Department of Experimental Radiation Oncology, The University of Texas MD Anderson Cancer Center, Houston, TX 77030, United States; Department of Experimental Radiation Oncology, The University of Texas MD Anderson Cancer Center, Houston, TX 77030, United States

## Abstract

The dynamic synthesis and removal of poly (ADP-ribose) (pADPr) by poly (ADP-ribose) polymerase 1 and poly (ADP-ribose) glycohydrolase (PARG), respectively, is essential for the maintenance of genome integrity, particularly during DNA replication. However, the precise role of the pADPr-binding chromatin remodeler chromodomain helicase DNA binding protein 1-like (CHD1L) in resolving endogenous DNA damage remains unclear. Here, we identified CHD1L as a critical modulator of Okazaki fragment maturation. We demonstrated that CHD1L loss was synthetic lethal with severe inhibition of PARG. This synthetic lethality stemmed from the toxic accumulation of pADPr specifically in S-phase cells, originating from unprocessed Okazaki fragment intermediates. Rescue experiments confirmed that both the ATPase and pADPr-binding macrodomain of CHD1L were indispensable for preventing this toxic accumulation. Mechanistically, quantitative chromatin proteomics revealed that CHD1L deficiency caused the persistent and aberrant retention of single-strand break (SSB) repair factors and others including the E3 ubiquitin ligases RNF114 and DTX3L/PARP9, challenging the previous models that CHD1L loss would limit chromatin accessibility of DNA repair factors. These findings establish a novel housekeeping function for CHD1L in facilitating the efficient turnover of DNA repair factors at replication-associated SSBs.

## Graphical Abstract

**Graphical Abstract:**
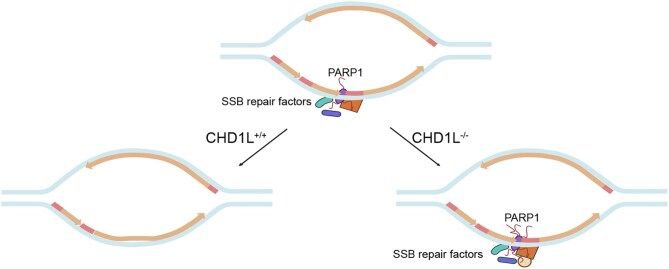

## Introduction

Genome integrity is maintained continuously via a dynamic signaling process involving the synthesis (PARylation) and removal (dePARylation) of poly (ADP-ribose) (pADPr) [1, 2]. This process is orchestrated by two key enzymes: poly (ADP-ribose) polymerase 1 (PARP1) and poly (ADP-ribose) glycohydrolase (PARG). Upon recognizing DNA strand breaks, PARP1 generates pADPr chains on itself and other acceptor proteins [3]. This rapid and localized pADPr accumulation serves as a signaling platform, which recruits downstream DNA damage response (DDR) factors to facilitate chromatin decompaction and subsequent DNA repair. The pADPr signaling is inherently transient and must be promptly removed to allow the completion of DNA repair and to restore normal chromatin function. The dePARylation is predominantly carried out by PARG [4]. The balance between PARylation and dePARylation is not only essential for proper DDR but also critical for cell survival [5–7]. Disruption of this balance, such as through the complete loss of PARG, can lead to the accumulation of unresolved repair intermediates and ultimately, cell death [8].

DNA replication during S-phase represents a period of heightened genome vulnerability, as the exposure of single-stranded DNA at the replication fork creates a hotspot for DNA damage. The lagging strand is synthesized discontinuously as a series of Okazaki fragments, a process that naturally generates a high density of transient single-strand breaks (SSBs) that must be efficiently processed and ligated. Normally, the coordinated processing and ligation/maturation of Okazaki fragments is carried out by FEN1 and LIG1. The sporadic unsuccessfully ligated Okazaki fragments would be sensed by PARP1, which promotes the synthesis of endogenous pADPr specifically in S-phase and recruits XRCC1/LIG3 to complete the ligation or maturation of Okazaki fragments [9]. The failure to timely remove S-phase pADPr, such as in the case of PARG inhibition, would lead to cell death [8]. More importantly, the failure to ligate Okazaki fragments increases pADPr in S-phase cells due to persistent SSBs and is a major source of replication stress. Indeed, the disruption of Okazaki fragment maturation not only sensitizes cells to PARP inhibitors (PARPis) [10, 11], but also to PARG inhibitors (PARGis) [8, 12, 13]. Therefore, the PARP1/PARG axis plays a crucial housekeeping role during S-phase to preserve the genome integrity.

Chromodomain helicase DNA binding protein 1-like (CHD1L), also known as ALC1, is an ATP-dependent chromatin-remodeling enzyme equipped with both an ATPase/helicase domain and a C-terminal macrodomain that recognizes and binds to pADPr [14]. The pADPr binding relieves an autoinhibitory conformation to activate the ATPase activity of this chromatin remodeler [15, 16]. CHD1L is recruited to sites of DNA damage in a PAR-dependent manner and remodels chromatin to facilitate the access of the lesion by downstream repair factors [17–19]. Multiple studies have shown that CHD1L is involved in base excision repair (BER) and SSB repair [14, 18, 20]. Together, these data support a model in which CHD1L acts as an early chromatin remodeler that transiently remodels nucleosomes to facilitate hand-off from PARP-dependent signaling to downstream repair factors. Furthermore, CHD1L is frequently amplified in several human cancers, where its overexpression is often correlated with poor prognosis, suggesting a role in promoting tumor cell survival under conditions of genomic stress [21–23].

The functional interplay between CHD1L and pADPr signaling has significant therapeutic implications, particularly concerning the use of PARPis. Several studies have established that the loss of CHD1L sensitizes cells to PARPi [24–28]. Although multiple mechanisms have been proposed, including the trapping of PARP1 or PARP2 and reduced chromatin accessibility for repair factors, the underlying basis remains incompletely defined. Notably, PARP1 facilitates CHD1L-dependent chromatin remodeling not only by generating pADPr to relieve ALC1 autoinhibition but also through its noncatalytic domains that mediate nucleosome binding and structural modulation, thereby positioning and stabilizing CHD1L for effective remodeling [29]. However, a significant gap remains in our understanding of CHD1L’s primary function in the absence of exogenous damage or PARP inhibition. It is not fully understood which specific endogenous DNA lesions require CHD1L for their resolution, particularly during unperturbed DNA replication, or how its function interplays with the PARylation/dePARylation cycle.

In this study, we investigated the relationship between CHD1L and pADPr signaling. We identified CHD1L as a key modulator of replication-associated DNA repair and found that its loss exacerbated cytotoxicity caused by severe PARG inhibition. We demonstrated that this synthetic lethality stemmed from the accumulation of pADPr specifically during S-phase, originating from unprocessed Okazaki fragment intermediates. Mechanistically, we showed that CHD1L loss led to the persistent and toxic association of SSB repair factors and others in chromatin, including the E3 ubiquitin ligase RNF114 and the DTX3L/PARP9 complex. Our findings revealed the novel function of CHD1L as a central modulator of Okazaki fragment maturation, which acts primarily to prevent the accumulation of toxic repair intermediates.

## Materials and methods

### Cell lines

HEK293A, HeLa, and U2OS cells were purchased from the American Type Culture Collection (ATCC) and cultured in Dulbecco’s modified Eagle’s medium (DMEM, corning) with 10% fetal bovine serum (Sigma). Knockout cells generated in HEK293A and HeLa cells were created with pLentiCRISPRv2 (Addgene, #52961) containing indicated guide RNA (gRNA) (Supplementary Table S1) as described previously [8]. All knockout cells were validated by western blotting and DNA sequencing (Supplementary Table S1). The HEK293A-derived *PARG*^hypo^, POLB KO, XRCC1 KO, LIG1 KO, APEX1 KO, TDP1 KO, and LIG3 KD cells were the same as those used in the previous studies [8, 30]. U2OS RNF114 KO were from Dr. Ivan Matic [31]. All cell lines used in this study were free of mycoplasma contamination.

### Immunofluorescence

U2OS and U2OS CHD1L KO cells were seeded into the glasses. After 24 h, cells were treated with dimethyl sulfoxide (DMSO) or PARGi for 4 h, and cells were released from PARGi treatment for 0.5, 1.5, or 4 h. After washing with PBS, cells were pre-extracted by PBS with 0.2% Triton X-100 on ice for 5 min, then fixed with 4% paraformaldehyde (PFA) at room temperature for 15 min. After fixation, cells were permeabilized with 0.5% Triton X-100 (in PBS) for 15 min and blocked with 4% BSA in PBS for 1 h. Cells were further incubated with the antibody against XRCC1 overnight at 4°C, then incubated with a secondary antibody the next day and counterstained with 4′,6-diamidino-2-phenylindole (DAPI). The stained slides were subjected to Nikon microscope to acquire images.

### Plasmid constructs

CHD1L (HsCD00821556) cDNA in pDONR221 vector was purchased from DNASU and subjected to mutagenesis using the QuikChange II Site-Directed Mutagenesis Kit following the manufacturer’s instructions. WT and mutant constructs were subsequently cloned into pLenti CMV Puro DEST (W118–1) (Addgene, #17452) or pMH-SFB (Addgene, #99391) by the GATEWAY cloning system. short hairpin RNA (shRNA) constructs (pGIPZ-based vector) targeting TDP1 (clone ID: V2LHS_174 999 and V2LHS_175 002) were obtained from Horizon Discovery Biosciences.

### Antibodies and inhibitors

Antibodies against CHD1L (13460S), PARP1 (9532S), and DTX3L (14795S) were purchased from Cell Signaling Technology. The antibody against pADPr (10 H) (AM80) was obtained from Millipore. The antibody against LIG3 (GTX70143) was from GeneTex. Antibodies against histone H3 (ab18521) and POLB (ab26343) were from Abcam. The antibody against TDP1 (sc-365674) was from Santa Cruz Biotechnology. Antibodies against PNKP (HPA006782), RNF114 (HPA021184), and β-Actin (A5316) were from Sigma. Antibody against PARP9 (17535-1-AP) was from Proteintech. Antibody against Mono-ADP-ribose was purchased from Bio-Rad (TZA020). PARG inhibitor PDD 00017273 (HY-108360) were obtained from MedChemExpress.

### Cell viability assays with CellTiter-Glo

To determine the sensitivity of cells to inhibitors, cells were seeded in 96-well plates. Twenty-four hours later, inhibitors with the indicated concentrations were added to the well. The highest concentration is 100 µM, followed by 3-fold serial dilutions across nine concentrations. The last one was DMSO. After 72 h, cell viability was measured by the CellTiter-Glo Luminescent Cell Viability Assay (G7573, Promega), according to the manufacturer’s instructions. All experiments were repeated at least three times or otherwise indicated.

### Clonogenic assay

The indicated cells were seeded on 12-well plates and treated with the indicated inhibitors for 7–14 days. Following washing with phosphate-buffered saline (PBS), cells were stained with crystal violet solution (HT90132, Sigma). The representative results were presented. All experiments were repeated at least three times or otherwise indicated.

### shRNA knockdown and reconstitution

The lenti-virus, used to infect cells, was generated either by pGIPZ vectors containing scrambled or gene-specific shRNAs or the expression construct with the packaging vector psPAX2, the envelope vector pMD2.G, and polyethyleneimine. After infection, cells were selected with puromycin for ∼5 days. The pooled cells, validated by Western blotting, were used in further experiments.

### CRISPR–Cas9-mediated stable knockout cells

The pLentiCRISPRv2 plasmids containing the gRNAs targeting the indicated genes were used to transfect cells with the aid of polyethyleneimine. Twenty-four hours later, these transfected cells were selected with puromycin for another 48 h. Cells survived puromycin selection were seeded into 96-well plates for single-cell cloning. The single clone KO cells were validated by western blotting and DNA sequencing. Knockout clone was generated with single gRNA targeting the indicated gene.

### Chromatin/soluble fractionation and western blotting

The chromatin/soluble fractionation was performed following the previous protocol [8, 30]. Briefly, trypsinized cells were resuspended in NETN buffer (20 mM Tris–HCl [pH 8.0], 1 mM EDTA, 100 mM NaCl, 0.5% NP-40, and 1 mM dithiothreitol (DTT)) containing proteinase inhibitors and 10 µM PARP/PARGis for 20 min on ice. After centrifugation, the supernatant (soluble fraction) was collected into fresh tubes. The pellets (the chromatin fractionation) were washed twice with NETN buffer. The 2 × Laemmli buffer was used to dilute the soluble and chromatin fractions. After being boiled at 95°C for 10 min, the samples were subjected to western blotting analysis. Briefly, after being separated by sodium dodecyl sulfate polyacrylamide gel electrophoresis (SDS–PAGE), samples were transferred to membranes and immunoblotted with the indicated antibodies. For each western blot, at least two biological replicates were performed, and the representative results were presented.

### Flow cytometry

Treatment of cells with emetine, POLA inhibitors (Aphidicolin, CD437, or Adarotene), PARGi, or double thymidine block was conducted the same as previous described [8]. Cells were then collected and fixed with pre-cold 70% ethanol, permeabilized with 0.5% Triton X-100 (in PBS) for 15 min. Cells were incubated with an antibody against pADPr overnight at 4°C, then stained with an Alexa Fluor 488 secondary antibody and FxCycle Violet Stain (1 μg/ml) (F10347, Thermofisher). The stained cells were subjected to flow cytometry analysis by Attune Flow cytometers (ThermoFisher). FlowJo software (v10.6.1) was used to analyze the acquired data. For each experiment, at least three biological replicates were performed, and the representative results were presented.

### Mass spectrometry analysis

The pellets from chromatin/soluble fractionation were resuspended in NETN buffer with 2% SDS. After being boiled at 95°C for 5 min, the samples were sonicated at 4°C (2-min cycles of 5 s on and 10 s off at 30% output power for a tip-probe sonicator), then subjected to reduction and alkylation. After centrifugation, the supernatant was subjected to acetone precipitation. After trypsin digestion, the tryptic peptides were cleaned up with C18 stagetips. Each sample has four biological replicates. The data acquisition was performed by data-independent acquisition (DIA) mode with LC gradient and mass spectrometry parameters as previously described [32].

The mass spectrometry raw files were processed using DIA-NN (version 1.9.1) with default parameters for DIA label-free quantification and searched against the human proteomics database from uniport (25 July 2023 updated, 82 089 proteins). Significance testing of log2-transformed intensities was performed with LIMMA on all peptides/proteins present in >70% of samples. Proteins with *P*-value ≤ 0.01 and a fold change cutoff of 1.5 were considered as significantly changed.

### Immunoprecipitation

CHD1L KO cells were transfected either with an empty vector or constructs encoding SFB-tagged CHD1L WT or mutants by polyethyleneimine. After 24 h, cells were treated with indicated chemicals with the same condition as previous study [8], then lysed with cold NETN buffer with sonication. After centrifugation, the supernatant was collected and incubated with S protein beads for 1 h at 4°C. The beads were washed three times with NETN buffer and boiled at 95°C for 10 min with 2 × Laemmli buffer. The elution was subjected to western blotting with the indicated antibodies.

## Results

### CHD1L loss exacerbates cytotoxicity and enhances S-phase pADPr accumulation induced by severe PARG inhibition

We showed previously that complete PARG inhibition led to cell lethality [8]. However, we were able to obtain viable clones with frameshift deletions when using sgRNAs targeting the N-terminus or the middle region of PARG, but not those targeting the C-terminal catalytic domain of PARG [8]. These and other results presented in our previous report indicate that these N-terminal or the middle region *PARG* KO cells are hypomorphic alleles. Therefore, we decide to refer them as *PARG*^hypo^ cells in this study. These *PARG*^hypo^ cells do not express full-length PARG and display dramatically reduced PARG activity [8]. However, they are viable [8] and suitable for further genetic studies, which is the reason we used these *PARG*^hypo^ cells in this study. Treating *PARG*^hypo^ cells with high doses of PARGi effectively mimics the complete loss of *PARG*, which led to cell lethality [8].

**Figure 1.**
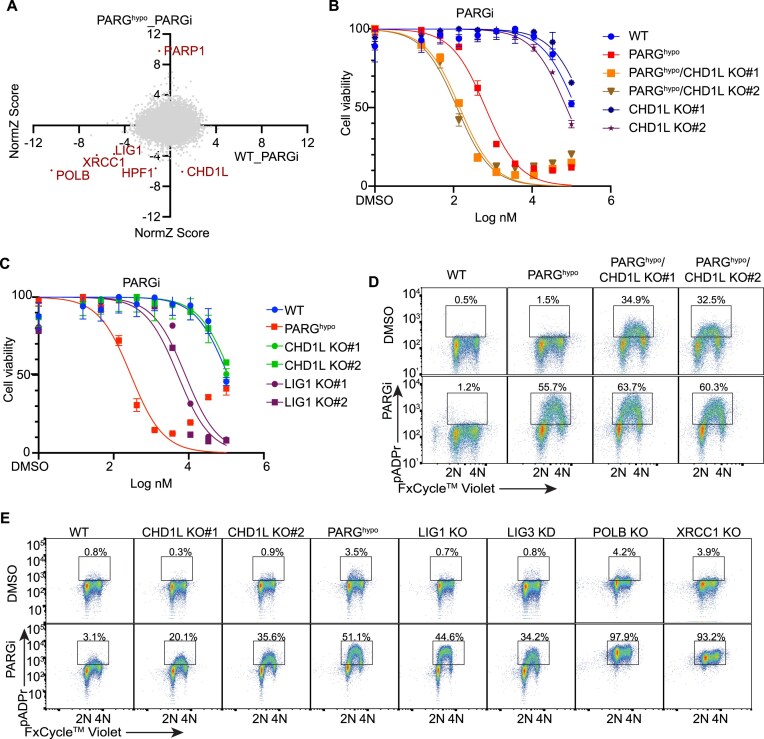
CHD1L loss induces S-phase–specific pADPr under PARG inhibition. (**A**) Replotted from Nie *et al*., 2024, eLife [8] to show CHD1L loss is synthetic lethal with PARG inhibition. (**B**) CHD1L loss sensitizes HEK293A *PARG*^hypo^ cells to PARGi, determined by CellTiter-Glo assay. Cells were treated with different doses of PARGi for 72 h. (**C**). The sensitivity to PARGi was measured by CellTiter-Glo assay in HEK293A WT, CHD1L KO, and LIG1 KO cells with different doses of PARGi treatment for 72 h. (**D**). Flow cytometry analysis of pADPr signaling in HEK293A *PARG*^hypo^ and *PARG*^hypo^ /CHD1L KO cells with PARGi treatment (10 µM, 4 h). A rectangular gate was drawn to highlight and quantify the percentage of cells with S-phase pADPr. Values represent the mean percentage of S-phase pADPr–positive cells. (**E**). Flow cytometry analysis of pADPr signaling in HEK293A WT, CHD1L KO, POLB KO, XRCC1 KO, LIG1 KO, and LIG3 knockdown cells with PARGi treatment (10 µM, 4 h).

FACS-based CRISPR screening results indicated CHD1L loss enhances the pADPr signal in the context of PARG loss or PARG inhibition [8]. To further investigate the role of CHD1L, we re-analyzed the cell viability-based screening data in both HEK293A WT and *PARG*^hypo^ cells treated with PARGi (Fig. 1A). Strikingly, CHD1L loss showed synthetic lethality exclusively in *PARG*^hypo^ cells but not in WT cells treated with PARGi (Fig. 1A). These data agree with the above-mentioned FACS-based screening results conducted with *PARG*^hypo^ cells, which suggest that the pADPr signaling further increased in *PARG*^hypo^ cells with CHD1L loss. Thus, CHD1L may function to suppress pADPr signaling during normal cell proliferation.

We noted that PARP1 loss was able to rescue cell lethality in *PARG*^hypo^ cells treated with PARGi (Fig. 1A), indicating that PARP1 is the major enzyme promoting pADPr during normal cell proliferation and that PARP1 and PARG counteract each other as we showed previously [8].

To validate the synthetic lethality relationship between PARG and CHD1L, we knocked out CHD1L in WT and *PARG*^hypo^ cells. Consistently, CHD1L KO increased the sensitivity of *PARG*^hypo^ cells, but not WT cells to PARGi (Fig. 1B). Previous reports show CHD1L is involved SSB repair, especially in BER [18, 26–28]. We included cell lines with canonical SSB repair gene deficiency, such as POLB KO, XRCC1 KO, LIG1 KO, and LIG3 KD cells, to assess the role of CHD1L in SSB repair (Fig. 1C and Supplementary Fig. S1A). Interestingly, while cell lines with SSB repair gene deficiency showed hypersensitivity to PARGi and PARPi, CHD1L KO cells were only sensitive to PARPi (Fig. 1C, and Supplementary Fig. S1A and B). To ensure these results were not specific to one cell type, we generated U2OS-derived CHD1L KO cells. As expected, U2OS-derived CHD1L KO cells did not display enhanced sensitivity to PARGi, but showed increased sensitivity to PARPi (Supplementary Fig. S1C and D), which is consistent with previous reports [24, 26].

It was puzzling that CHD1L KO cells were not sensitive to PARGi. One likely explanation is that PARGi is not very effective in completely inhibiting PARG activity and CHD1L depletion only exacerbates cytotoxicity induced by severe PARG inhibition, i.e. in the case of *PARG*^hypo^ cells treated with PARGi. We decided to further test this possibility by examining pADPr signaling.

The ligation of Okazaki fragments and downstream SSB repair pathways are known to be crucial for the cellular response to PARGi. Disruption of these processes results in the accumulation of pADPr, either specifically in S-phase or throughout all cell cycle phases, under PARGi treatment [8]. 

Given the potential roles of CHD1L in suppressing pADPr signaling as mentioned above, we hypothesized that CHD1L loss would lead to the abnormal accumulation of pADPr specifically in S-phase cells.

We showed that S-phase pADPr did not increase dramatically in control WT cells even with PARGi treatment (Fig. 1D). However, S-phase pADPr increased noticeably in *PARG*^hypo^ cells treated with PARGi (Fig. 1D). These observations agree with our hypothesis above that PARGi is not very effective in completely inhibiting PARG activity. We were only able to observe significant S-phase pADPr in *PARG*^hypo^ cells treated with PARGi.

As expected, CHD1L loss caused the accumulation of S-phase pADPr in *PARG*^hypo^ cells, even without PARGi treatment (Fig. 1D). Furthermore, CHD1L loss resulted in mild S-phase pADPr accumulation in HEK293A WT cells with PARGi treatment, similar to those seen with the loss of genes such as LIG1 and LIG3 (Fig. 1E), which are involved in Okazaki fragment ligation or maturation. In contrast, loss of canonical SSB repair genes (e.g. POLB and XRCC1) led to increased pADPr signaling throughout the cell cycle (Fig. 1E), which agrees with our previous observations [8]. Notably, loss of APEX1, which is believed to act downstream of CHD1L in BER pathway [25, 28], did not affect pADPr signaling with or without PARGi treatment (Supplementary Fig. S1E). Moreover, this S-phase–specific pADPr induction was not cell-type-specific, as it was also observed in U2OS-derived CHD1L KO cells (Supplementary Fig. S1F). Together, these data strongly suggest that CHD1L has a role in Okazaki fragment maturation and its loss would result in enhanced S-phase specific pADPr signaling.

### CHD1L suppresses pADPr formation induced by unligated Okazaki fragments

**Figure 2.**
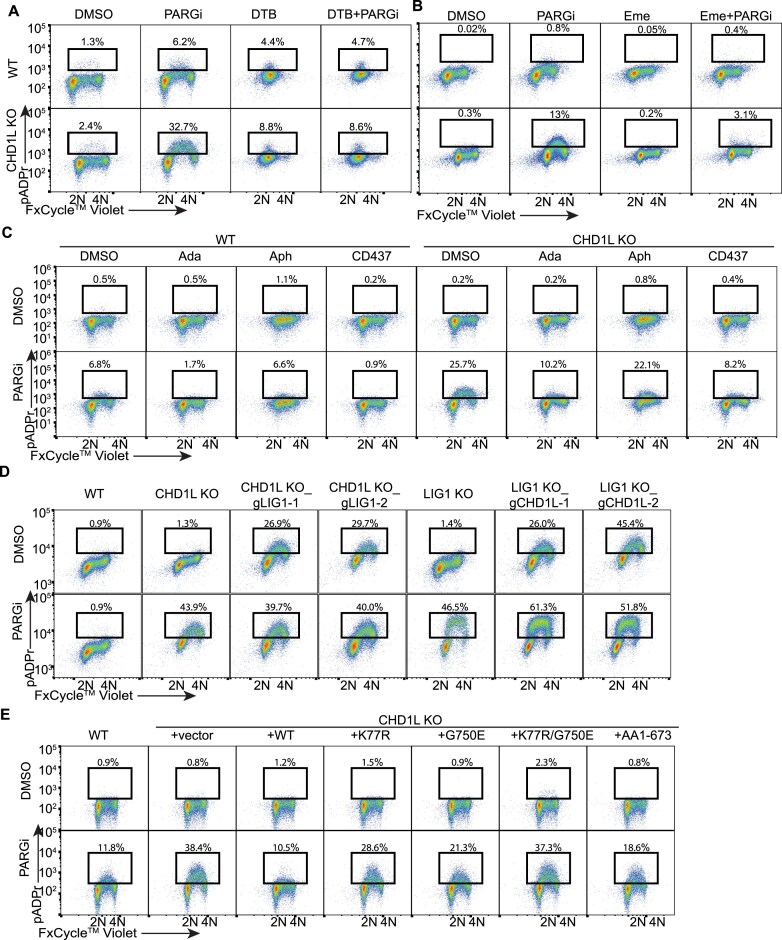
S-phase pADPr induced by CHD1L loss originates from unligated Okazaki fragment intermediates. (**A**) Flow cytometry analysis of pADPr signaling in cells synchronized with DTB. After DTB, cells were treated with PARGi (10 µM) for 4 h. A rectangular gate was drawn to highlight and quantify the percentage of cells with S-phase pADPr. Values represent the mean percentage of S-phase pADPr–positive cells. (**B**) Emetine-treated (2 µM, 90 min) cells followed an additional 4 h PARGi (10 µM) treatment were stained with anti-pADPr antibody to detect the S-phase pADPr signaling. (**C**) The S-phase pADPr signaling in cells pre-treated with POLA inhibitors including Aphidicolin (Aph) (400 nM), CD437 (200 nM), or Adarotene (Ada) (800 nM) for 16 h, then 10 µM PARGi for another 4 h. (**D**) CHD1L KO cells were infected with lentiviruses expressing gRNAs targeting LIG1, and LIG1 KO cells were transduced with gRNAs targeting CHD1L to generate pooled double knockout populations. Flow cytometry analysis of pADPr signaling levels was performed in the indicated cells with or without PARGi treatment (10 µM, 4 h). (**E**) Flow cytometry analysis of pADPr signaling in HEK293A WT, CHD1L KO and cells reconstituted with either full-length CHD1L, ATPase domain mutation (K77R), macrodomain mutation (G750E), both or the truncation without macrodomain (AA1-673). Cells were treated with 10 µM PARGi for 4 h.

The S-phase–specific pADPr is believed to originate from unligated Okazaki fragments [8, 9]. To ensure that this is still the case under CHD1L loss, we synchronized cells at the G1/S boundary using a double thymidine block (DTB) protocol. As expected, the DTB successfully suppressed the S-phase pADPr in CHD1L KO cells (Fig. 2A). Furthermore, treatment with emetine, a translation inhibitor that inhibits nascent protein synthesis and causes replication forks to stall [8, 9], significantly abolished the S-phase pADPr signal in CHD1L KO cells (Fig. 2B), which again links the pADPr accumulation with active replication and replication-associated DNA damage.

We reasoned that if the pADPr signal induced by CHD1L loss arose from lesions on the lagging strand, inhibiting the initiation of Okazaki fragments should abrogate this signal. Indeed, pre-treating cells with inhibitors of DNA polymerase α (Aphidicolin, CD437, or Adarotene) substantially reduced the S-phase pADPr signal in CHD1L KO cells treated with PARGi (Fig. 2C). These data together provide strong evidence that unligated Okazaki fragment intermediates are likely the primary source of S-phase pADPr in CHD1L KO cells treated with PARGi.

To exclude the possibility that DNA damage arising outside of S-phase, such as nicks or gaps caused by defective BER, contributes to the accumulation of pADPr in S-phase in CHD1L KO cells, we perturbed Okazaki fragment ligation by knocking out LIG1 or LIG3 (Supplementary Fig. S2A). LIG1 is the primary ligase responsible for canonical Okazaki fragment joining, whereas LIG3 mediates the ligation of a subset of Okazaki fragments that escape the canonical pathway. In the BER pathway, LIG3 predominantly ligates single-strand nicks, while LIG1 has been implicated in long-patch BER.

Interestingly, loss of CHD1L and LIG3 did not increased pADPr as measured by western blot (WB) or FACS (Supplementary Fig. S2B and C), suggesting that CHD1L functions in the same pathway as LIG3 during S-phase. Notably, CHD1L/LIG1 double KO cells exhibited markedly reduced proliferation, indicative of a potential synthetic lethal interaction (data not shown). Furthermore, Furthermore, CHD1L/LIG1 double knockout cells displayed S-phase–specific pADPr accumulation even in the absence of PARGi treatment, which was further enhanced upon PARG inhibition (Fig. 2D and Supplementary Fig. S2D).

Although loss of LIG1 is expected to generate increased levels of unligated Okazaki fragments during S-phase, pADPr remained below the detection limit without additional PARG inhibition. One possible explanation is that PARP1 senses unligated Okazaki fragments and facilities the recruitment of repair factors such as LIG3, while PARG is simultaneously recruited to remove pADPr in a rapid and dynamic manner. Loss of CHD1L may disrupt this coordinated process, impairing the function of LIG3 and thereby promoting the accumulation of unligated Okazaki fragments.

To confirm that this phenotype is a direct result of CHD1L’s function, we performed rescue experiments. Reconstitution of CHD1L KO cells with wild-type CHD1L completely abolished the S-phase pADPr signaling (Fig. 2E). CHD1L has two key functional domains: two N-terminal ATPase/helicase domains and a C-terminal pADPr-binding macrodomain [14, 15]. We found that constructs with mutations in either the ATPase domain (K77R) or the macrodomain (G750E) were unable to suppress pADPr. These data demonstrate that both the chromatin remodeling activity and the ability to recognize and bind to pADPr are indispensable for CHD1L’s function in resolving Okazaki fragment intermediates.

### CHD1L loss leads to persistent chromatin association of SSB repair factors

**Figure 3.**
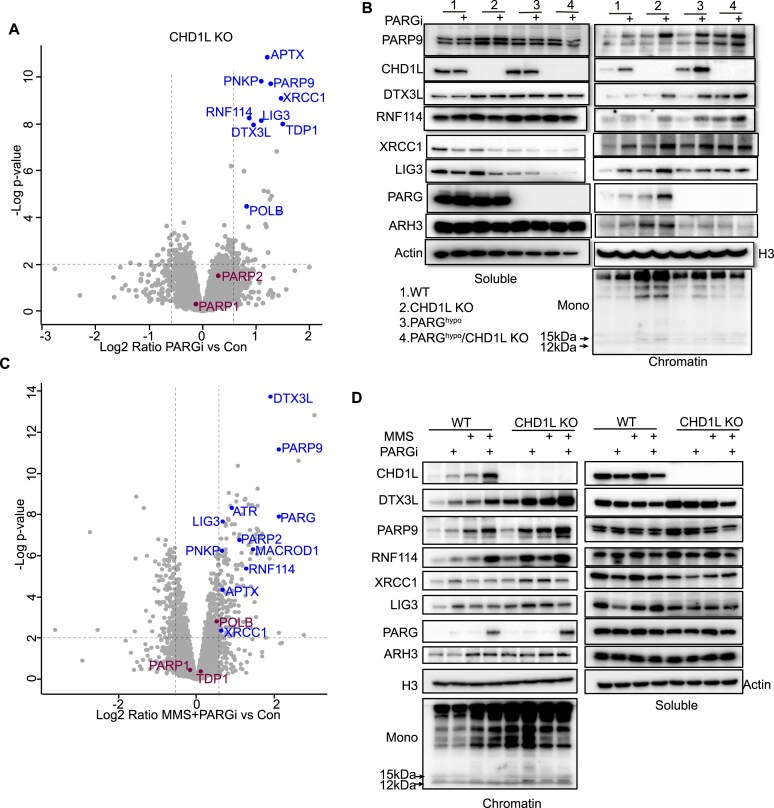
CHD1L loss induces prolonged chromatin binding of SSB repair proteins. (**A**) Proteomic analysis of chromatin isolated from HEK293A CHD1L KO cells with/without PARGi (10 µM, 4 h) treatment. Volcano plot shows the differentially changed proteins. Each dot denotes a protein. The significantly changed proteins were determined by −log *P*-value ≥ 2 and absolute value of log2 ratio ≥ 1.5, as indicated by the dashed lines. (**B**) Immunoblots of soluble and chromatin-bound protein levels in different knockout cells treated with/without PARGi. (**C**) Proteomic analysis of chromatin isolated from HEK293A CHD1L KO cells with/without MMS (0.01% MMS for 30 min) and PARGi (10 µM, 4 h) treatment. Volcano plot shows the differentially changed proteins. Each dot denotes a protein. The significantly changed proteins were determined by −log *P*-value ≥ 2 and absolute value of log2 Ratio ≥ 1.5, as indicated by the dashed lines. (**D**) Immunoblots of soluble and chromatin-bound protein levels in HEK293A WT and CHD1L KO cells treated with MMS (0.01% MMS for 30 min), PARGi (10 µM, 4 h), or both.

To further characterize the consequences of CHD1L loss, we performed quantitative proteomic analysis of chromatin-bound proteins in HEK293A CHD1L KO cells with or without PARGi treatment. Previous studies suggest that CHD1L loss reduces chromatin accessibility and consequently limits the binding of repair factors to chromatin [27, 28]. However, we observed enrichment of numerous SSB repair factors, including XRCC1, LIG3, APTX, PNKP, POLB, and TDP1 on chromatin (Fig. 3A and Supplementary Table S2). The enrichment of TDP1 was particularly noteworthy, as CHD1L prevents replication-fork collapse caused by camptothecin (CPT), a topoisomerase I (TOP1) poison that traps TOP1 on DNA, which promotes the formation of TOP1 cleavage complexes (TOP1cc) that can be removed by TDP1 [20]. CPT treatment induces DNA replication-dependent DNA damage [30], while TDP1 loss alone also led to more unligated DNA replication intermediates, as indicated by S-phase pADPr signaling in TDP1 KO cells treated with PARGi (Supplementary Fig. S3A). Depleting TDP1 did not further increase S-phase pADPr signaling in CHD1L KO cells following PARGi treatment (Supplementary Fig. S3B), suggesting CHD1L has a more general involvement in DNA replication compared to TDP1. The enrichment of TDP1 on chromatin further supported the idea that CHD1L is involved in resolving Okazaki fragment intermediates and/or the modulation of replication fork progression.

Unexpectedly, we did not observe the enrichment of PARP1/2 on chromatin, even though they are responsible for sensing unligated Okazaki fragments [9]. It is possible that PARP1 chromatin association may be transient and could not be easily detected by our fractionation assay. Notably, among those proteins that were enriched on chromatin are the recently identified PARylation/MARylation-binding E3 ubiquitin ligases RNF114 and DTX3L/PARP9 complex (Fig. 3A). RNF114 is recruited to DNA lesions, binds to MARylated proteins and ubiquitinates MARylated proteins (MARUbylation), and also extends K11 polyubiquitin on sites of MARUbylation, and inhibition of RNF114 has been shown to induce the trapping of PARP1 [31, 33–36]. The DTX3L/PARP9 complex functions in both cytosol and nucleus, regulating interferon signaling and DNA damage repair by ubiquitinating protein substrates [37–40]. Recent studies have shown DTX3L/PARP9 can ubiquitinate single-stranded nucleic acids and ADP-ribosyl modification on nucleic acids [41–43] and is recruited into DNA lesions in a pADPr-dependent manner [44]. Indeed, in agreement with our proteomics analysis, CHD1L loss enhanced the chromatin association of RNF114 and DTX3L/PARP9 in both WT and *PARG*^hypo^ cells following PARGi treatment (Fig. 3B). Notably, even without PARGi treatment, marked accumulation of RNF114 and DTX3L/PARP9 on chromatin was observed when CHD1L was knocked out in *PARG*^hypo^ cells (Fig. 3B). Interestingly, analogous to PARP trapping induced by PARPis, PARG was also trapped on chromatin upon PARGi in both WT and CHD1L KO cells. Notably, loss of CHD1L alone was sufficient to induce PARG trapping, which is further enhanced by PARGi (Fig. 3B). Moreover, a similar pattern was observed for XRCC1 and LIG3. In contrast, ARH3, another macrodomain-containing protein, accumulated on chromatin only upon CHD1L loss but not following PARG inhibition (Fig. 3B).

To determine whether the selective accumulation of DTX3L/PARP9 and RNF114, both implicated in histone MARylation, is driven by their macrodomain-mediated binding or their functional association with histones, we further examined histone MARylation levels (Fig. 3B). Unexpectedly, loss of CHD1L led to increased histone MARylation, which was further enhanced upon PARG inhibition. However, we were unable to clearly distinguish whether the enrichment of DTX3L/PARP9 and RNF114 is primarily mediated by their macrodomain-dependent recognition of ADP-ribosylation or by their involvement in histone modification. It is therefore likely that both macrodomain-dependent binding and histone MARylation contribute to the accumulation of DTX3L/PARP9 and RNF114 on chromatin.

The chromatin accumulation of the proteins mentioned above was observed in normal proliferating cells without any exogenous DNA damage. To further investigate whether CHD1L loss would limit the chromatin association of repair factors under DNA damage conditions, we performed chromatin proteomics using cells treated with methyl methanesulfonate (MMS) and PARGi to maximize the retention of pADPr signaling (Fig. 3C and Supplementary Table S3). Consistently, multiple repair factors were enriched on chromatin. In addition to BER and SSB repair factors, we also observed the enhanced chromatin association of PARG, MACROD1, ATR, and PARP2 but not PARP1 or TDP1. Consistently, the augmented chromatin association of RNF114 and DTX3L/PARP9 was observed in CHD1L KO cells following MMS treatment, and this enrichment was further enhanced with the addition of PARGi treatment (Fig. 3D). Notably, analogous to PARP trapping induced by MMS in combination with PARPis, PARG was also robustly retained on chromatin upon MMS and PARGi treatment in both WT and CHD1L KO cells (Fig. 3D). Moreover, similar results were obtained in U2OS-derived CHD1L KO cells (Supplementary Fig. S3C).

Interestingly, knockout of DTX3L/PARP9 did not affect the chromatin retention of CHD1L or RNF114 in either WT or *PARG*^hypo^ cells treated with PARGi, MMS or both (Supplementary Fig. S4A and B). Consistent with chromatin retention results, knockout of DTX3L/PARP9 did not make cells more sensitive to PARGi in either WT or *PARG*^hypo^ cells (Supplementary Fig. S4C).

**Figure 4.**
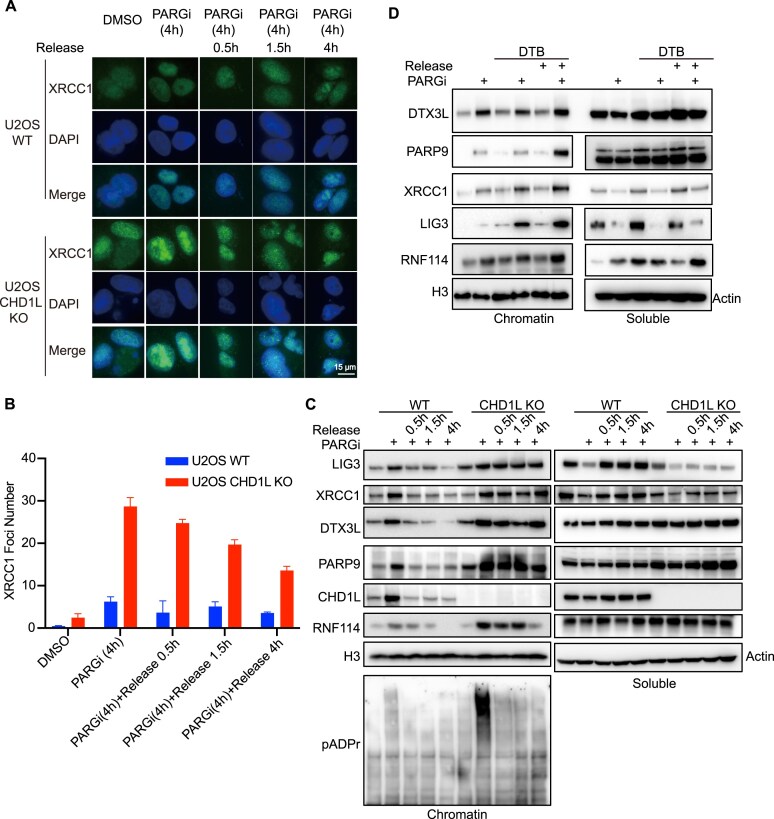
CHD1L loss disrupts the dissociation of SSB repair proteins from chromatin. (**A**) XRCC1 immunostaining was performed in pre-extracted WT and CHD1L KO U2OS cells treated with DMSO or PARGi for 4 h, followed by release for 0.5, 1.5, or 4 h. Representative images are shown. (**B**) Quantification of XRCC1 foci number in different groups are graphed from panel (A). The mean and s.d. of *n* = 3 independent experiments are shown. (**C**) Immunoblots of soluble and chromatin-bound proteins were conducted using extracts prepared from WT and CHD1L KO cells treated with DMSO or PARGi for 4 h, followed by release for 0.5, 1.5, or 4 h. (**D**) Immunoblots of soluble and chromatin-bound proteins in CHD1L KO cells. CHD1L KO cells were synchronized by DTB. Cells were either maintained in DTB or released from DTB and treated with/without 10 µM PARGi for 4 h.

To further characterize the consequences of CHD1L loss, specifically, whether loss of CHD1L affected the dissociation of factors from chromatin, we examined the chromatin-bound protein levels in pre-extracted cells released from PARGi treatment at different time points (Fig. 4A and B). Consistent with our previous results, combined CHD1L loss and PARGi treatment led to significant accumulation of XRRC1 on chromatin. As expected, XRCC1 rapidly dissociated from chromatin upon PARGi removal in WT cells, while XRCC1 remained retained on chromatin even 4 hours after PARGi removal in CHD1L KO cells (Fig. 4A and B).

To determine whether other key factors exhibited a similar pattern and to minimize technical variability, we performed chromatin fractionation followed by WB under the same condition (Fig. 4C). Consistent with the immunofluorescence results, XRCC1 remained persistently associated with chromatin after PARGi removal in CHD1L KO cells. Moreover, a similar pattern was observed for additional factors, including LIG3, DTX3L/PARP9, and RNF114. Interestingly, this sustained chromatin association following PARGi removal may not depend on pADPr level, as pADPr rapidly decreased to the basal level of CHD1L-deficient cells upon inhibitor withdrawal. Notably, these findings are consistent with a previous study by Blessing *et al*. [26], which reported that CHD1L loss delayed the chromatin dissociation of PARP2 and XRCC1 following laser-induced DNA damage. Together, these results suggested CHD1L facilitate the dissociation/turnover of key factors on chromatin.

We have previously shown that CHD1L suppresses pADPr formation induced by unligated Okazaki fragments. To further support a mechanistic role of CHD1L specifically during S-phase and at unligated Okazaki fragments, we performed chromatin fractionation followed by WB after DTB and release to assess chromatin-associated protein levels (Fig. 4D). Following DTB, cells are enriched in early S-phase, and although DTB may induce replication stress, we still observed a detectable level of chromatin accumulation upon PARGi treatment. Notably, this accumulation became more pronounced after release from DTB (Fig. 4D). Token together, these data suggested CHD1L plays a critical role in the process of PARP1-dependent Okazaki fragment maturation.

Taken together, these results suggest that the aberrant accumulation of these factors is a specific consequence of CHD1L loss, both in normal proliferating cells and under DNA damaging conditions. These data indicate that CHD1L acts as a primary coordinator to facilitate the efficient turnover or resolution of these DNA repair and signaling proteins.

### CHD1L plays an important role in cellular response to DNA damage and PARPi by regulating chromatin retention of factors involved in SSB repair

**Figure 5.**
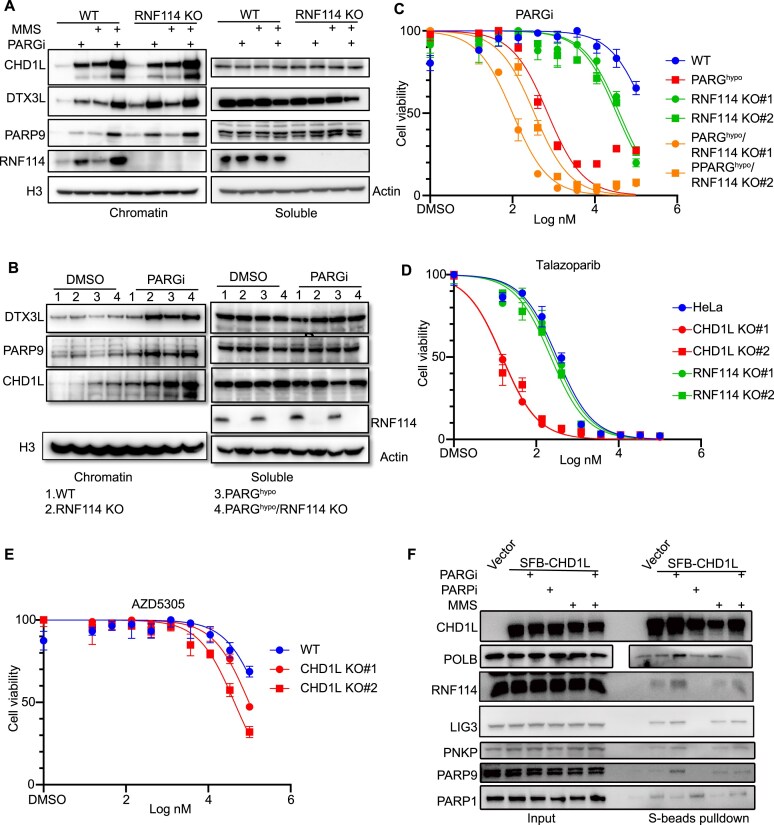
CHD1L regulates the chromatin binding of RNF114 and DTX3L/PARP9. (**A**) Immunoblots of soluble and chromatin-bound protein levels in HEK293A WT and RNF114 KO cells treated with MMS (0.01% MMS for 30 min), PARGi (10 µM, 4 h) or both. (**B**) Immunoblots of soluble and chromatin-bound protein levels in different knockout cells treated with/without PARGi (10 µM, 4 h). (**C**) The sensitivity to PARGi was measured by CellTiter-Glo assay in HEK293A WT, RNF114 KO, PARGhypo, and PARGhypo/RNF114 KO cells with different doses of PARGi treatment for 72 h. (**D**) HeLa WT cells, CHD1L KO, and RNF114 cells were treated with different doses of talazoparib for 72 h. Cell viability was determined by the CellTiter-Glo assay. (**E**) The sensitivity to AZD5305 was measured by CellTiter-Glo assay in HEK293A WT and CHD1L KO cells with different doses of AZD5305 treatment for 72 h. (**F**) HEK293A CHD1L KO cells were either transfected with an empty vector or construct encoding SFB-CHD1L, treated with PARGi (10 µM, 4 h), PARPi (10 µM, 4 h), MMS (0.01% MMS for 30 min), or PARGi + MMS, then were subjected to immunoprecipitation (IP) with S-beads. The blots were conducted with the indicated antibodies.

The persistent chromatin binding of RNF114 in the absence of CHD1L implied an intimate functional link between these proteins. Therefore, we investigated the potential role of RNF114 in the phenotypes observed in CHD1L KO cells. While CHD1L loss increased the chromatin association of RNF114 and DTX3L/PARP9 in response to treatment with PARGi, MMS, or both (Fig. 3D), the effects of RNF114 loss were different. RNF114 loss only modestly enhanced the chromatin association of DTX3L/PARP9, but not CHD1L, following PARGi treatment (Fig. 5A and B). Unexpectedly, RNF114 loss slightly decreased the chromatin association of CHD1L and DTX3L/PARP9 under MMS treatment (Fig. 5A). Consistently, RNF114 loss did not significantly affect the chromatin association of these proteins in *PARG*^hypo^ cells (Fig. 5B). Similar results were obtained in U2OS-derived RNF114 KO cells (Supplementary Fig. S5A). These results suggest that RNF114 may act downstream of or in concert with CHD1L, but RNF114 appears to have a relatively modest role in these processes.

These findings point to CHD1L as a central regulator of chromatin association for SSB repair proteins and/or factors involved in Okazaki fragment maturation. To further test this working hypothesis, we examined the sensitivity to PARGi in the absence of one or more of these regulatory proteins. Similar to CHD1L loss, RNF114 loss modestly sensitized cells to PARGi in *PARG*^hypo^ cells (Fig. 5C), which is consistent with a previous report [33]. However, RNF114 loss did not lead to the accumulation of S-phase pADPr in *PARG*^hypo^ cells (Supplementary Fig. S5B), indicating a distinct mechanism of action. Next, we tested the cellular sensitivity to MMS. Loss of RNF114 did not make cells more sensitive to MMS in either HEK293A or HeLa background, whereas CHD1L loss did (Supplementary Fig. S5C and D). Recent studies have highlighted that the presence of unrepaired replication gaps is a more direct cause of sensitivity to PARPi than the traditionally cited DSBs [10, 11, 28]. To further demonstrate that CHD1L, but not RNF114, is involved in Okazaki fragment maturation, we tested the sensitivity of CHD1L KO or RNF114 KO cells to PARPis such as Olaparib and Talazoparib. CHD1L loss sensitized cells to PARPi in both HEK293A and HeLa background, but RNF114 loss did not (Fig. 5D, and Supplementary Fig. S5E and F). Moreover, CHD1L loss modestly sensitized cells to PARP1-specific inhibitor, AZD5305, while RNF114 loss failed to do so (Fig. 5E and Supplementary Fig. S5G). Additionally, we found that CHD1L interacted with RNF114, DTX3L/PARP9, and several SSB repair factors (Fig. 5F). Taken together, these results supported a model in which CHD1L is recruited to replication-associated SSBs, where it facilitates the timely dissociation of RNF114, DTX3L/PARP9, and SSB repair factors from chromatin, thus preventing the formation of unrepaired replication gaps/nicks.

## Discussion

In this study, we uncovered a previously unrecognized role of CHD1L in maintaining replication integrity through the maturation of Okazaki fragments. Specifically, CHD1L deficiency led to the accumulation of S-phase pADPr (Fig. 1D and E), which was traced to unligated Okazaki fragment intermediates (Fig. 2A–D). At the mechanistic level, CHD1L loss induced aberrant chromatin accumulation of SSB repair proteins and/or factors involved in Okazaki fragment maturation under normal proliferation and DNA damage conditions (Figs 3 and 4). These results are contradictory to previous assumption that CHD1L loss would limit chromatin accessibility of DNA repair factors [27, 28]. Our data supported a direct role of CHD1L in the removal or turnover of these DNA replication and repair factors on chromatin, but not the previous models which favor an indirect role of CHD1L in these processes via its chromatin remodeling function. Under normal conditions, unligated Okazaki fragments are recognized by PARP1, which deposits pADPr to recruit CHD1L and DNA replication and repair factors. CHD1L facilitates the release or turnover of these DNA replication and repair factors and prevents their persistent retention on chromatin. In the absence of CHD1L, unligated Okazaki fragments persist, leading to sustained pADPr accumulation and aberrant chromatin association of DNA replication and repair factors.

During normal DNA replication, the unligated Okazaki fragments activate PARP1 to generate S-phase pADPr, which recruits XRCC1/LIG3 to process unligated Okazaki fragments [9]. However, this process is tightly controlled, in part via the actions of PARG and CHD1L. Indeed, we showed previously that complete inhibition of PARG leads to persistent accumulation of S-phase pADPr, which eventually results in cell death [8]. Here, we showed that CHD1L facilitates Okazaki fragments maturation and its loss increases S-phase pADPr. Structurally, the requirement of both the ATPase and macrodomain of CHD1L to suppress S-phase pADPr underscore its dual role in recognizing the PAR signal and executing a physical remodeling function. Mechanically, the increased S-phase pADPr in CHD1L-deficient cells is likely due to the persistent chromatin association of many DNA replication and repair factors. This process is exacerbated by the toxic effects of PARG inhibition, as PARG activity is essential for the removal of S-phase pADPr during replication [1, 4, 8]. These findings refine our understanding of the roles of CHD1L in DNA damage response. While previous studies have implicated CHD1L in BER and SSB repair pathways, our results pinpoint a specialized, essential housekeeping function during unperturbed DNA replication.

Interestingly, previous reports suggested that CHD1L loss leads to reduced chromatin accessibility, thereby limiting the chromatin binding of repair factors [27, 28]. Here, we propose that the primary function of CHD1L in this context is not merely to create an open chromatin state for the access of DNA replication and repair factors, but more critically, to facilitate the efficient turnover and dissociation of these DNA replication and repair machinery upon ligation. This hypothesis is derived from our unbiased proteomics data, which revealed the persistent chromatin association of DNA replication and repair factors in CHD1L deficient cells. In the absence of CHD1L, these factors remain bound to the chromatin. The situation is exacerbated when PARG is inhibited, since the pADPr signals that mark these sites could not be erased, effectively locking these unresolved replication and repair factors in place and leading to replication catastrophe and cell death. This working hypothesis can explain the synthetic lethality between CHD1L loss and PARG inhibition.

The accumulation of RNF114 in CHD1L-deficient cells is of particular interest, as RNF114 has been implicated in regulating PARP1 activity and reducing PARP1 trapping at DNA lesions [31, 33–36]. Notably, CHD1L associates with RNF114 and loss of CHD1L markedly enhances the chromatin association of RNF114 even in the absence of exogenous DNA damage. However, these data cannot establish a strict linear pathway in which CHD1L functions upstream of RNF114. Although RNF114 retention was enhanced upon CHD1L loss, RNF114 depletion did not phenocopy the S-phase pADPr accumulation or PARPi sensitivity observed in cells with CHD1L loss. Therefore, these findings are more consistent with a model in which CHD1L regulates the broader turnover of PARylation-associated repair and signaling factors, including RNF114. Whether RNF114 acts downstream of CHD1L, in parallel with CHD1L, or as part of a broader PARylation-dependent chromatin-retention network remains to be determined.

Importantly, CHD1L loss, but not RNF114 loss, sensitizes cells to PARPis. We showed that CHD1L loss promotes PARPi sensitivity through the accumulation of unligated Okazaki fragments, as replication gaps may be a more direct cause of sensitivity to PARPi than the traditionally cited DSBs [10, 11, 28], thereby defining CHD1L as a novel determinant of PARPi response in cancer cells. However, CHD1L loss differentially sensitizes cells to various PARPis. It appears to CHD1L loss significantly sensitize cells to Talazoparib, modestly to Olaparib, and only mildly to AZD5305. These differences may reflect whether these PARPis trap PARP1/2 and other replication/repair proteins, which may rely more on CHD1L function. Additionally, a potent PARP1-specific inhibitor such as AZD5305 may significantly reduce pADPr and therefore prohibit the recruitment and function of CHD1L in these processes. More broadly, these differential responses may also arise from the distinct trapping potency and PARP isoform selectivity of these inhibitors. Potent PARP trappers, such as Talazoparib, induce persistent PARP–DNA complexes and associated repair intermediates that likely require efficient chromatin remodeling and factor turnover, thereby increasing reliance on CHD1L. In contrast, inhibitors with reduced trapping activity or increased PARP1 selectivity, such as AZD5305, may partially bypass this requirement by limiting both PARP trapping and PARP2-dependent contributions. Together, these observations suggest that both PARP trapping and isoform selectivity contribute to the context-dependent sensitivity of CHD1L-deficient cells to PARPis.

Many questions remain unresolved. First, when is CHD1L recruited to chromatin or sites of DNA damage? Previous studies showed that CHD1L functions after the removal of the damaged base by DNA glycosylase but before APEX1 incision and acts to enhance APEX1’s ability to cleave nucleosome-embedded AP sites [25, 28]. However, in the classical SSB repair pathway, PARP1 senses SSBs and synthesizes pADPr after APEX1 incision [45]. Given that CHD1L’s function is pADPr-dependent, it remains unclear whether CHD1L is recruited before or after APEX1 function. We did not observe APEX1 chromatin retention in our proteomics data during normal proliferation or under DNA damage conditions. Of course, in this study we did not perform more sensitive assays such as live cell imaging experiments to determine whether CHD1L is recruited before or simultaneously with these DNA replication and repair factors identified by our proteomics analysis.

Second, it remains puzzling that several DNA “clean-up” proteins, such as PNKP and APTX, were retained in the chromatin upon CHD1L loss. In theory, Okazaki fragment maturation does not require the function of PNKP or APTX. This raises the question whether CHD1L may have additional SSB repair related functions in normal proliferating cells beyond its involvement in facilitating Okazaki fragment maturation. One possibility is that the retention of these factors reflects their association with a broader spectrum of replication-associated DNA intermediates. Consistent with this idea, previous studies have identified PNKP, APTX and related factors as proteins enriched at nascent DNA, suggesting that they may function more broadly at sites of ongoing DNA synthesis [46]. Another possibility is that the retention of these factors reflects a broader response to elevated pADPr signaling. In addition, as our analyses were performed in asynchronous cell populations, we cannot exclude the possibility that the observed accumulation partly reflects defects in other DNA repair processes upon CHD1L loss. Together, these observations suggest that the retention of these “clean-up” factors may represent both direct and indirect consequences of CHD1L deficiency.

Third, another key question is how CHD1L facilitates the release of these DNA replication and repair factors from chromatin. Especially, the initial retention of repair factors requires pADPr, but their subsequent persistence on chromatin may be, at least in part, independent of pADPr levels. CHD1L is a chromatin remodeler; its loss should limit chromatin accessibility of these replication and repair proteins. However, our results do not support this point of view. As a matter of fact, there may be very limited or no chromatin structure at the sites of Okazaki fragments. Therefore, we favor the model that CHD1L may promote the turnover or the release of these factors from DNA, either directly or indirectly. Since Both CHD1L and these replication and repair factors are pADPr binding proteins, a potential explanation is that CHD1L loss induces PARP1 accumulation and more pADPr on DNA, which lead to chromatin accumulation of these pADPr binding factors. The only caveat is that we did not identify or observe PARP1/2 retention under these conditions. Last, it remains unknown how RNF114 and DTX3L/PARP9 function and what their physiological substrates are. Loss of RNF114 sensitized cells to PARGi, consistent the previous report showing that RNF114 inhibitor nimbolide synergizes with PARGi in UWB1 cells [33]. However, the underlying mechanism remains poorly understood, particularly with respect to the potential functional interplay between RNF114 and CHD1L. Notably, in contrast to RNF114, loss of DTX3L/PARP9 does not sensitize cells to PARG inhibition, suggesting that these factors may play distinct and non-redundant roles in this pathway. Answering these questions will further elucidate this CHD1L-dependent pathway and may open new avenues for developing targeted cancer therapies.

A key unresolved issue is the causal relationship between the persistent chromatin retention of DNA replication and repair factors and the synthetic lethality observed upon combined CHD1L loss and PARG inhibition. While our data support a strong correlation between these two phenomena, a direct mechanistic linkage remains to be formally established. It remains possible that synthetic lethality arises primarily from perturbed pADPr homeostasis or replication stress, rather than being directly driven by the chromatin “locking” of repair factors.

Importantly, disentangling these possibilities is further complicated by the intrinsic coupling between pADPr signaling, repair factor recruitment, and chromatin turnover. Although pADPr-dependent recruitment contributes to the initial accumulation of repair proteins, sustained chromatin retention cannot be fully explained by pADPr levels alone. Consistently, pADPr levels can rapidly decline upon PARGi withdrawal, whereas a subset of repair factors remains chromatin-associated, supporting a model in which CHD1L facilitates the dissociation and turnover of repair complexes. Moreover, increased pADPr does not necessarily correlate with the presence of unrepaired DNA breaks, as no substantial increase of DNA damage is detected in PARG^hypo^ cells treated with short-term PARG inhibition which display markedly increased pADPr levels [8]. These observations argue against a model in which CHD1L loss primarily impairs completion of repair reactions and instead favor a scenario in which repair is largely completed but factor release from chromatin is delayed.

A related consideration applies to the interpretation of the persistent chromatin retention of factors in Fig. 3. In this experiment, PARGi was used to stabilize transient pADPr signals and enhance detection of repair factor recruitment. This design inevitably complicates the separation of CHD1L-dependent effects from pADPr-driven chromatin occupancy. Although CHD1L loss alone alters the chromatin association of selected repair factors, PARG inhibition further amplifies these signals in both WT and CHD1L-deficient cells, indicating that both CHD1L status and pADPr availability contribute to the observed proteomic signatures. Therefore, the chromatin occupancy patterns likely represent an integrated output of CHD1L function and pADPr signaling rather than a strictly linear pathway.

Together, these considerations suggest that CHD1L operates within a highly interconnected network governing replication-associated repair factor dynamics, in which pADPr signaling, factor recruitment, and chromatin turnover are tightly coupled and not readily deconvolved under current experimental constraints.

In summary, our study reveals a novel function of CHD1L in the maintenance of genome integrity by facilitating Okazaki fragment maturation. We hypothesize that CHD1L prevents the toxic accumulation of unresolved DNA replication and repair complexes, thereby linking its function directly to the fidelity of DNA replication.

## Funding

This work was conducted at the University of Texas MD Anderson Cancer, and was supported in part by institutional funds, the Sheila Abrams Prenowitz and Donald Morton Prenowitz Distinguished University Chair in Cancer Research, and NIH/NCI CA274234. J.C. also received support from NIH/NCI (CA275712 and CA278758). Funding to pay the Open Access publication charges for this article was provided by the internal funding.

## Supplementary Material

gkag606_Supplemental_Files

## Data Availability

All proteomics data in this study are present in the manuscript or the Supplemental Table. The acquired MS/MS raw data in this study have been deposited into the MassIVE dataset and can be accessed at: ftp://massive-ftp.ucsd.edu/v10/MSV000098826/.

## Notes

Present address: Furong Laboratory, Central South University, Changsha, Hunan 410078, China
