# Supplementary material for: CHD1L maintains genome integrity by facilitating okazaki fragment maturation
Source: Nucleic Acids Res. 2026 Jun 22;54(12):gkag606. doi: 10.1093/nar/gkag606 (PMC13284710; doi:10.1093/nar/gkag606)
Supplement: gkag606_Supplemental_Files [file gkag606_supplemental_files.zip › Supplementary information_05012026.docx]

**Supplemental figure legend**

**Figure S1. CHD1L loss sensitizes cells to PARPi.**

**A:** The sensitivity to PARGi was measured by CellTiter-Glo assay in WT, CHD1L KO, POLB KO, XRCC1 KO, LIG1 KO and LIG3 knockdown HEK293A cells with different doses of PARGi treatment for 72 hours. **B:** The cell viability of HEK293A WT, CHD1L KO, POLB KO, XRCC1 KO, LIG1 KO and LIG3 knockdown was measured by the CellTiter-Glo assay. Cells were treated with different doses of olaparib (left) or talazoparib (right) for 72 hours. **C:** The sensitivity to PARGi in U2OS WT and CHD1L KO cells was determined by CellTiter-Glo assay. Cells were treated with different doses of PARGi for 72 hours. **D:** Representative images and results (left) of clonogenic assays conducted using U2OS WT and CHD1L KO cells with the indicated doses of olaparib (up) or talazoparib (bottom) treatment for 14 days, and quantification of crystal violet staining assay (right). **E:** Flow cytometry analysis of pADPr signaling in HEK293A WT, CHD1L KO and APEX1 KO cells treated with PARGi (10 µM, 4 hours). **F:** Flow cytometry analysis of pADPr signaling in U2OS WT and CHD1L KO cells treated with PARGi (10 µM, 4 hours).

**Figure S2. CHD1L cooperates with LIG1 to regulate the Okazaki fragment maturation.**

**A:** Cells were infected with lentiviruses expressing the indicated gRNAs to generate pooled double knockout populations. Knockout efficiency was validated by Western blotting using antibodies against the corresponding proteins. **B:** Flow cytometry analysis of pADPr signaling levels was performed in CHD1L/LIG3 double knock out cells with or without PARG inhibitor treatment (10 µM, 4 hours). **C-D:** Immunoblots of pADPr levels in CHD1L/LIG3 double knock out cells (**C**) or CHD1L/LIG1 double knock out cells (**D**) with/without PARGi treatment.

**Figure S3. CHD1L affects the chromatin binding of RNF114 and DTX3L/PARP9**

**A:** Flow cytometry analysis of pADPr signaling in HEK293A WT and TDP1 KO cells treated with PARGi (10 µM, 4 hours). A rectangular gate was drawn to highlight and quantify the percentage of cells with S-phase pADPr. Values represent the mean percentage of S phase pADPr–positive cells. **B:** Flow cytometry analysis of pADPr signaling in HEK293A CHD1L KO cells with TDP1 knockdown treated with PARGi (10 µM, 4 hours). shRNA knockdown efficiency was confirmed by the immunoblot of TDP1. **C:** Immunoblots of soluble and chromatin-bound protein levels in U2OS WT and CHD1L KO cells treated with MMS (0.01% MMS for 30 min), PARGi (10 µM, 4 hours) or both.

**Figure S4. DTX3L has no impact on the chromatin binding of CHD1L or RNF114**

**A:** Immunoblots of soluble and chromatin-bound protein levels in HEK293A WT and DTX3L/PARP9 DKO cells treated with MMS (0.01% MMS for 30 min), PARGi (10 µM, 4 hours) or both. **B:** Immunoblots of soluble and chromatin-bound protein levels in different knockout cells treated with/without PARGi (10 µM, 4 hours). **C:** The sensitivity to PARGi was measured by the CellTiter-Glo assay in HEK293A WT, DTX3L/PARP9 DKO, *PARG*^hypo^, and *PARG*^hypo^ /DTX3L/PARP9 DKO cells with different doses of PARGi treatment for 72 hours.

**Figure S5. CHD1L loss contributes to cellular sensitivity to PARPi and MMS.**

**A:** Immunoblots of soluble and chromatin-bound protein levels in U2OS WT and RNF114 KO cells treated with MMS (0.01% MMS for 30 min), PARGi (10 µM, 4 hours) or both. **B:** Flow cytometry analysis of pADPr signaling in HEK293A WT, RNF114 KO, *PARG*^hypo^ and *PARG*^hypo^/RNF114 KO cells with PARGi treatment (10 µM, 4 hours). A rectangular gate was drawn to highlight and quantify the percentage of cells with S-phase pADPr. Values represent the mean percentage of S phase pADPr–positive cells. **C:** The sensitivity to MMS in HEK293A WT, RNF114 KO and *PARG*^hypo^ cells was measured by the CellTiter-Glo assay. Cells were treated with different doses of the reagent for 72 hours. **D:** The sensitivity to MMS in HeLa WT, CHD1L KO and RNF114 KO cells. **E:** The sensitivity to Talazoparib in HEK293A WT, RNF114 KO and *PARG*^hypo^ cells. **F:** The sensitivity to olaparib in HeLa WT, CHD1L KO and RNF114 KO cells. **G:** The sensitivity to AZD5305 in HEK293A WT and RNF114 KO cells.

**Table S1.** The gRNA sequences used to generate KO cell lines and the sequencing results.

**Table S2.** The DIA-NN outputs of chromatin fraction of CHD1L KO cells treated with/without PARGi (10 µM, 4 hours), related to **Figure 3A**.

**Table S3.** The DIA-NN outputs of chromatin fraction of CHD1L KO cells treated with/without MMS (0.01% MMS for 30 min)+PARGi (10 µM, 4 hours), related to **Figure 3B**.
