# Supplementary figures and images for: CHD1L maintains genome integrity by facilitating okazaki fragment maturation
Source: Nucleic Acids Res. 2026 Jun 22;54(12):gkag606. doi: 10.1093/nar/gkag606 (PMC13284710; doi:10.1093/nar/gkag606)

**Fig. S1**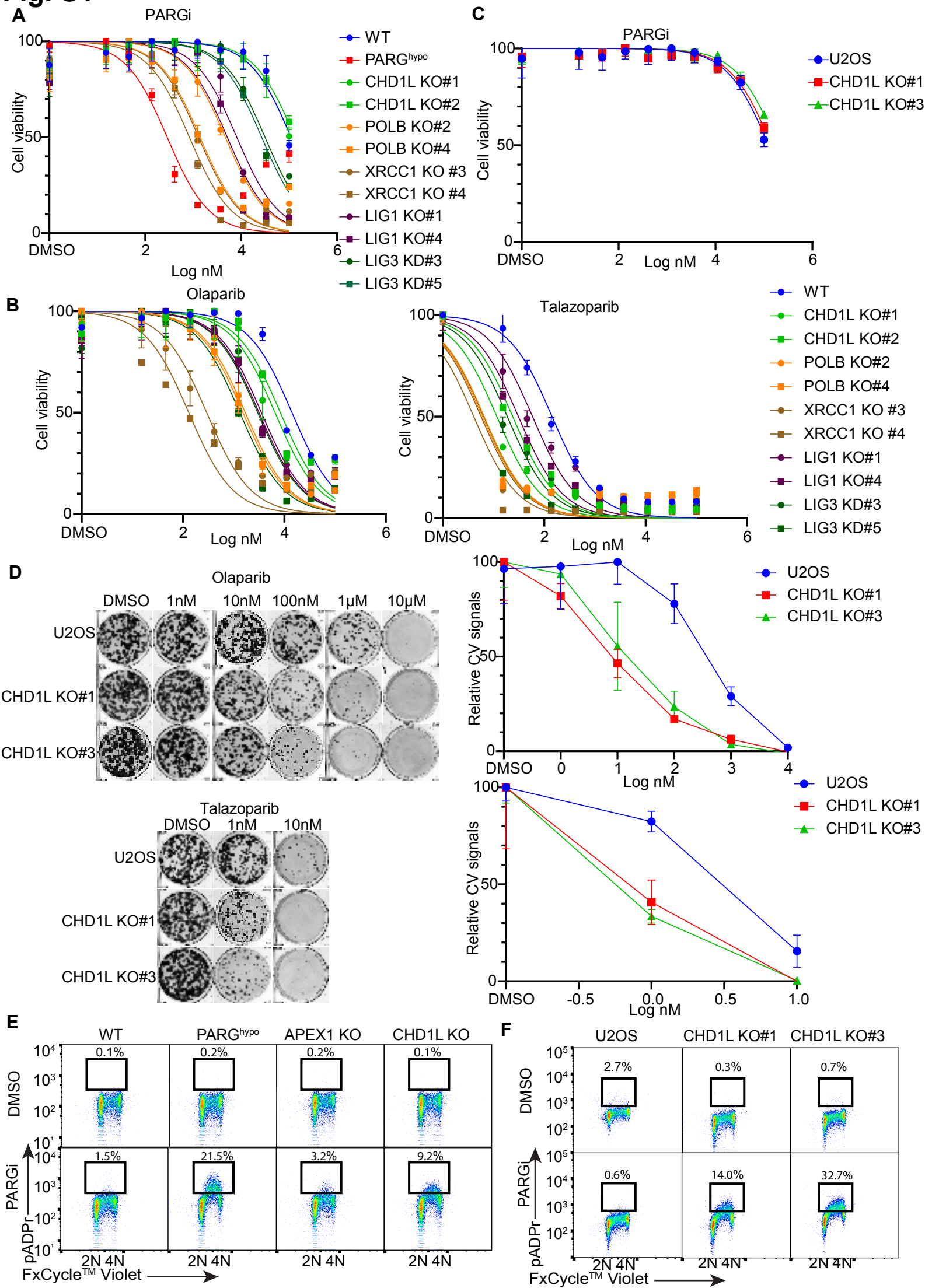

Supplement: gkag606_Supplemental_Files [file gkag606_supplemental_files.zip › Figure S1.pdf]

# Fig. S2

**A**

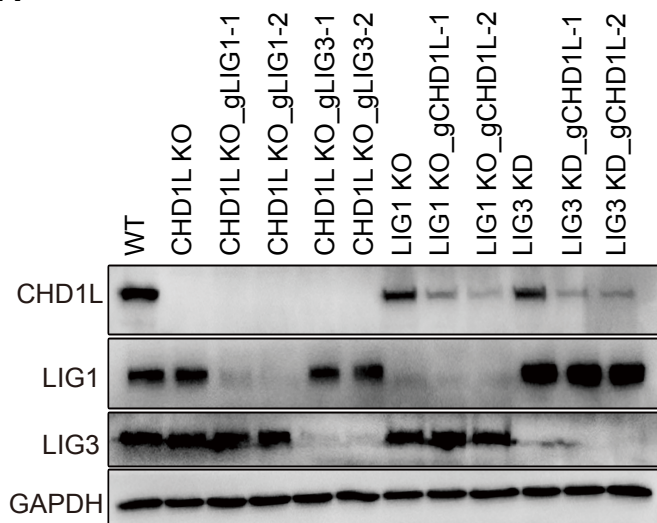

**C**

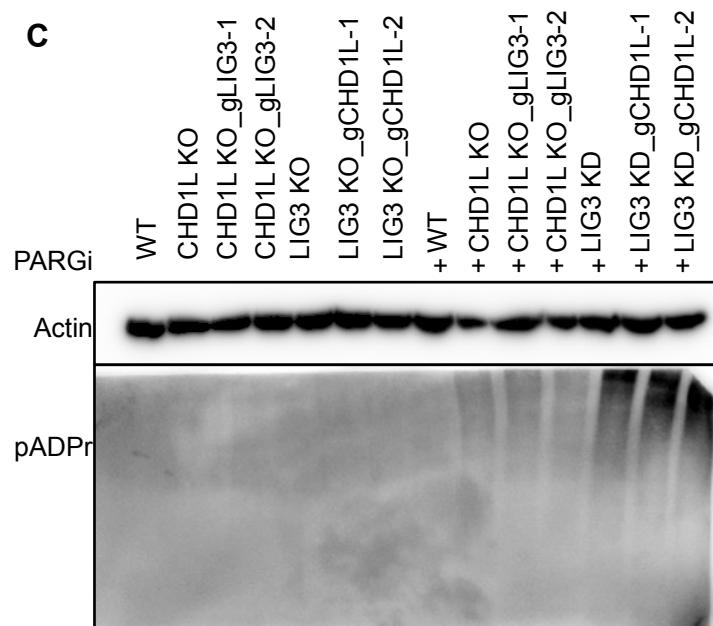

**B**

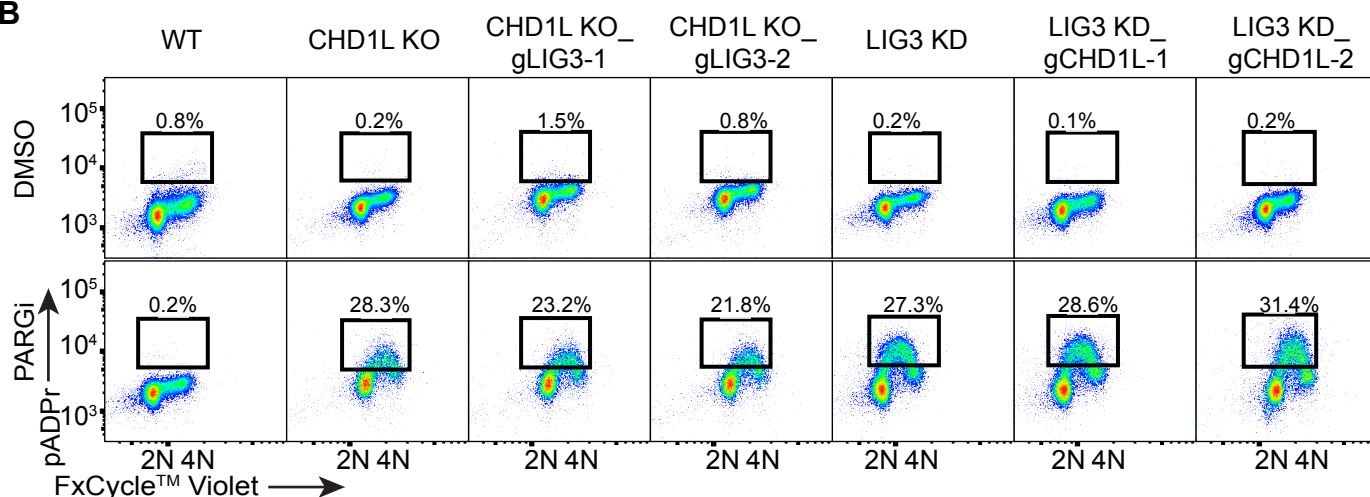

**D**

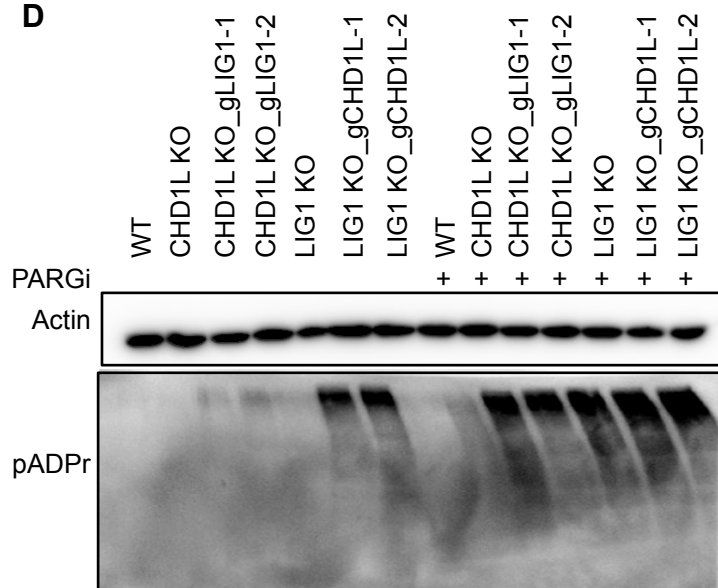

Supplement: gkag606_Supplemental_Files [file gkag606_supplemental_files.zip › Figure S2.pdf]

**Fig. S3**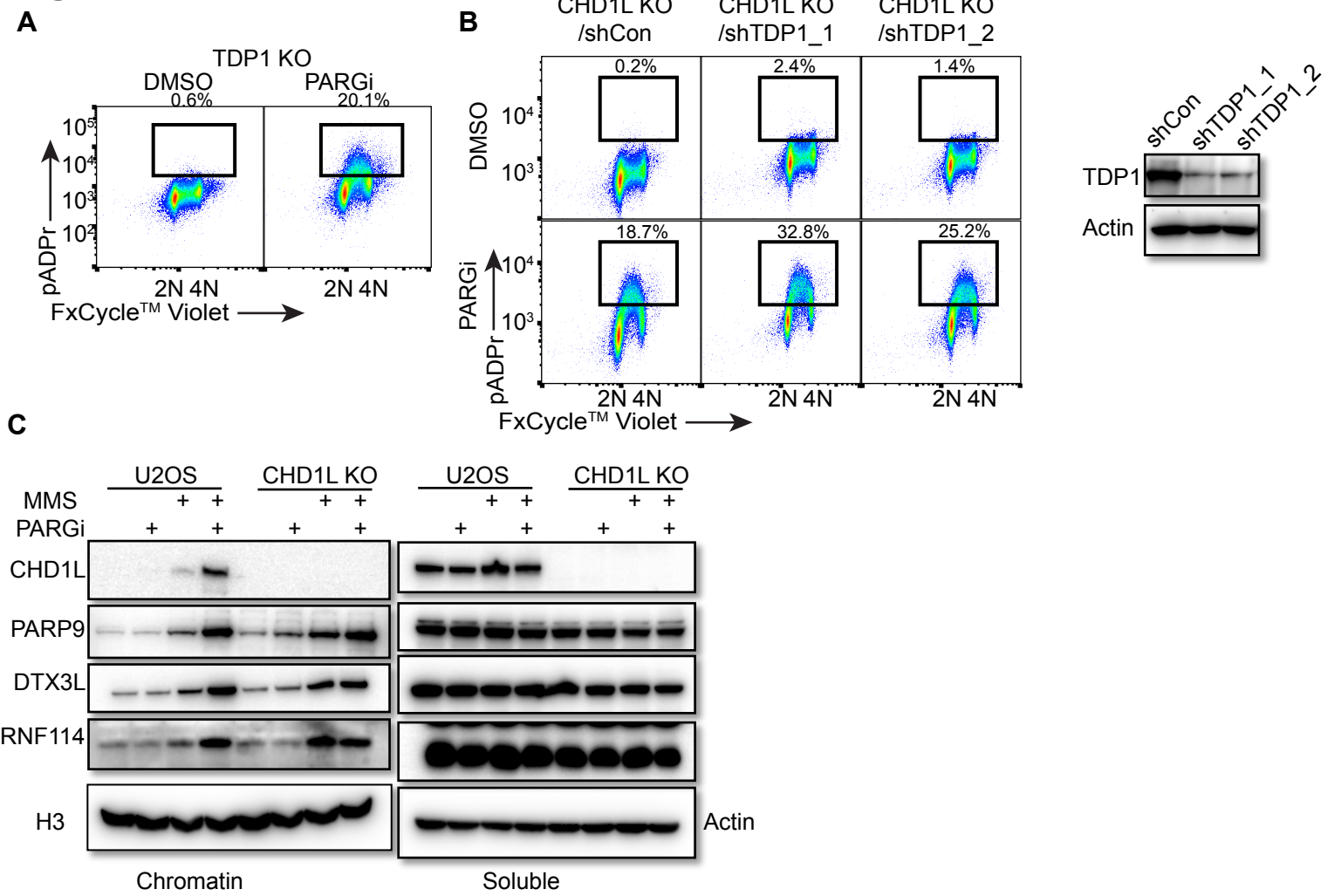

Supplement: gkag606_Supplemental_Files [file gkag606_supplemental_files.zip › Figure S3.pdf]

**Fig. S4****A**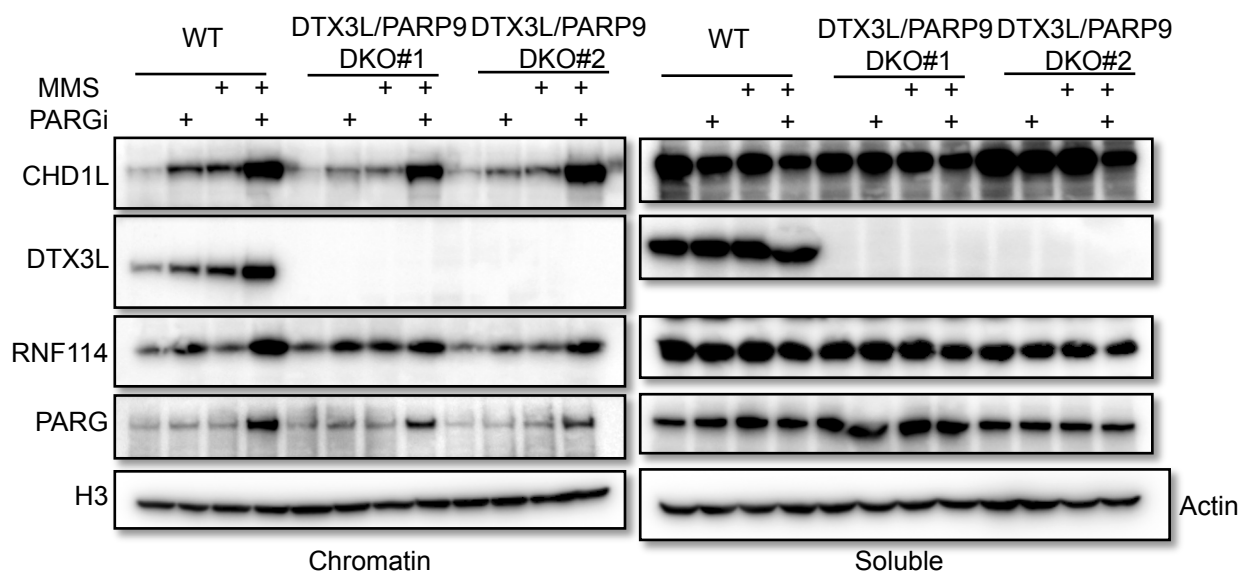**B**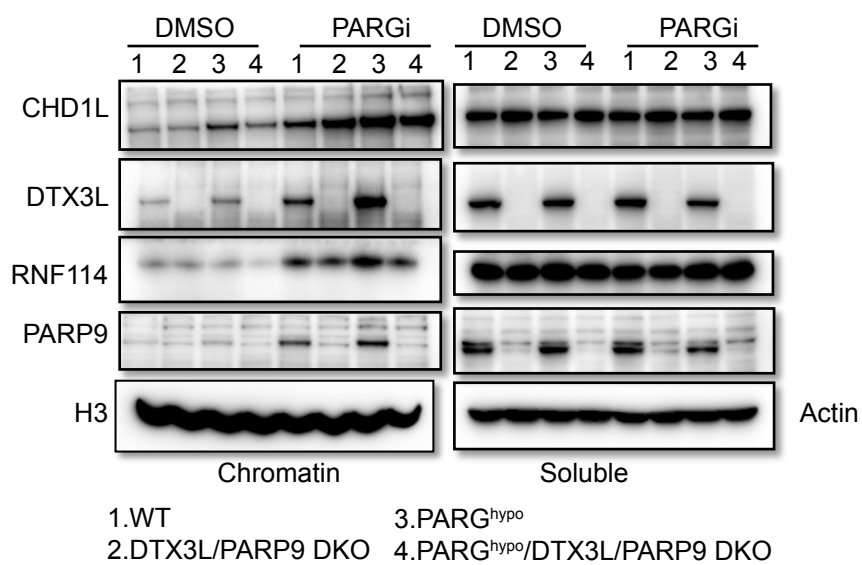**C**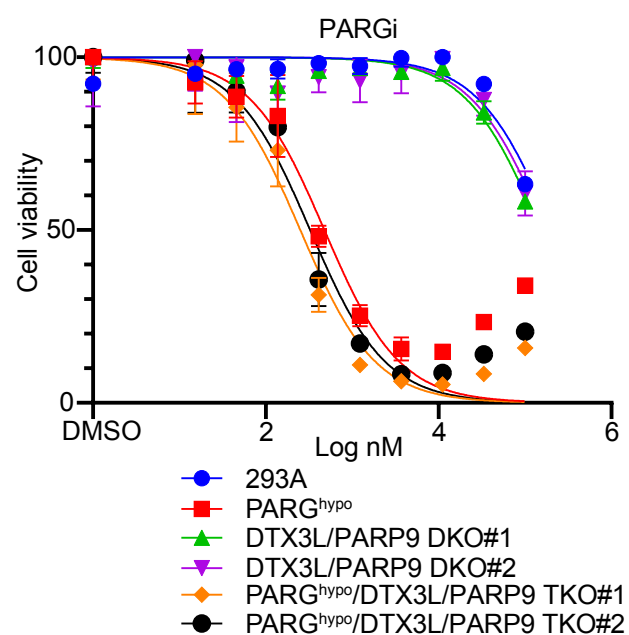

Supplement: gkag606_Supplemental_Files [file gkag606_supplemental_files.zip › Figure S4.pdf]

# Fig. S5

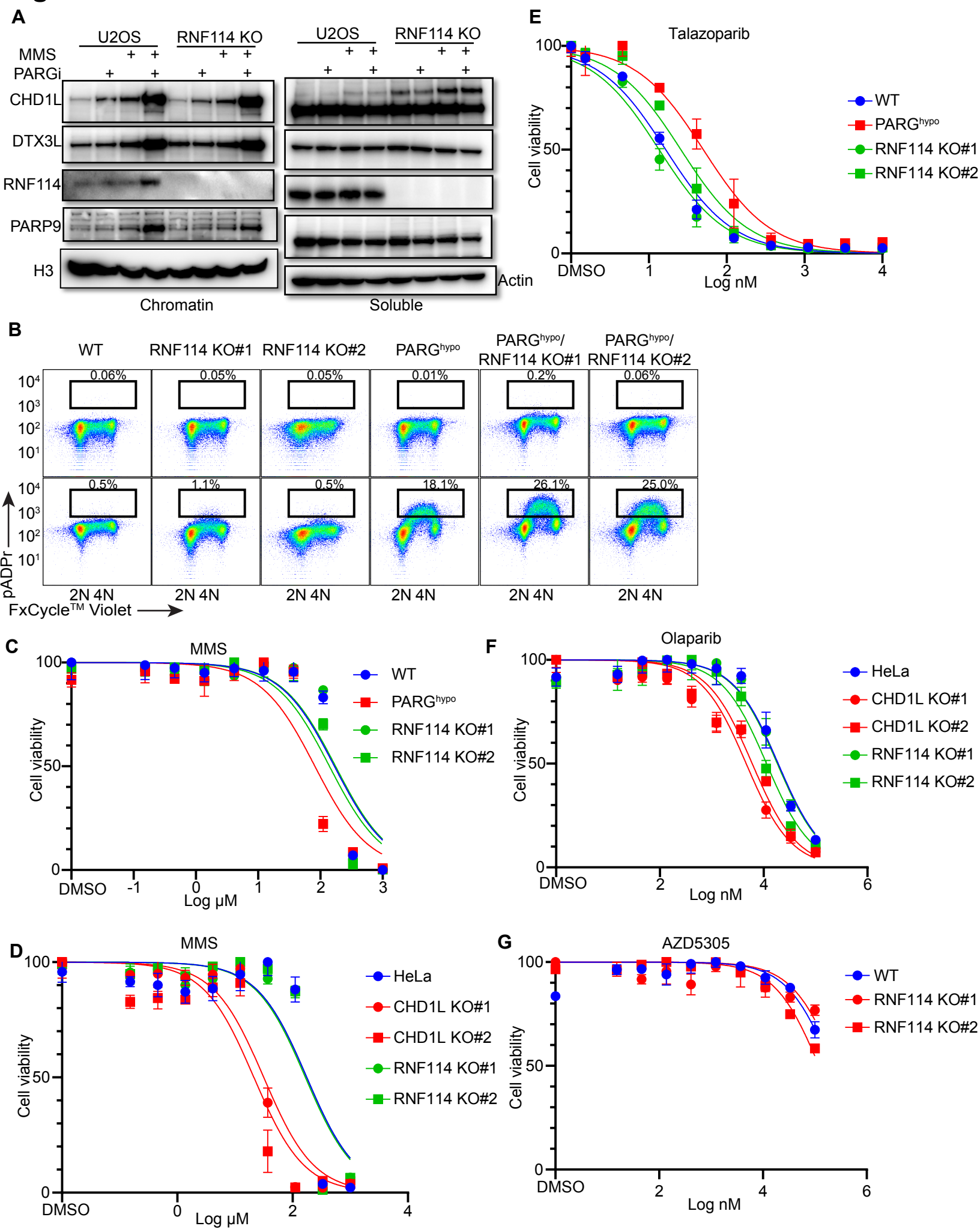

Supplement: gkag606_Supplemental_Files [file gkag606_supplemental_files.zip › Figure S5.pdf]
